# Alcohol consumption and risky sexual behaviors among fishers in Elmina in Ghana

**DOI:** 10.1186/s12889-023-16239-w

**Published:** 2023-07-11

**Authors:** Frank Kyei-Arthur, Sylvester Kyei-Gyamfi

**Affiliations:** 1 Department of Environment and Public Health, University of Environment and Sustainable Development, Somanya, Ghana; 2 Department of Children, Ministry of Gender, Children and Social Protection, Accra, Ghana

**Keywords:** Alcohol consumption, Risky sexual behavior, Condom use, Elmina, Ghana

## Abstract

**Background:**

Alcohol consumption is part of human social behavior and constitutes a routine part of social life in many countries. Prior studies have found over-indulgence of fishers in alcohol in fishing communities. The study uses the Alcohol Myopia Theory (AMT) to analyze and explain the phenomena of fishers engaging in sex after alcohol consumption, and condom use with sexual partner(s) after alcohol consumption. The study investigated alcohol consumption, predictors of alcohol consumption, and the reasons for drinking alcohol among fishers. It also examined fishers’ engagement in sex after alcohol consumption, the use of condoms with sexual partners after drinking alcohol, and predictors of the use of condoms with sexual partners after drinking alcohol.

**Methods:**

A cross-sectional convergent parallel mixed-method design was used to study 385 fishers in Elmina. Also, two focus group discussions were conducted among male and female fishers. Descriptive statistics were used to analyze the quantitative data, while the qualitative data was analyzed thematically.

**Results:**

Generally, 59.2% of participants indulged in alcohol consumption. Most male participants (70.6%) indulged in alcohol consumption than female participants (48.5%). Also, 48.5% of participants indulged in binge alcohol consumption, while 38.1% indulged in moderate alcohol consumption. The predictors of alcohol consumption were sex, religion, and type of fishing occupation. Participants identified consuming alcohol to kill loneliness and boredom, to forget family and work-related issues, and to have fun as reasons why fishers consume alcohol. Sixty-four percent of participants have ever engaged in sexual intercourse after consuming alcohol in the past 12 months. However, 70% of participants did not use a condom the last time they had sex after drinking alcohol. Only ethnicity of participants predicted their use of a condom the last time they had sex after drinking alcohol. The primary reasons for the non-use of condoms were do not like using condoms (37.9%), forgetting to use a condom (33.0%), and had sex with a trusted regular partner (15.5%).

**Conclusions:**

This study demonstrated that alcohol consumption was prevalent among fishers, especially among male fishers, which contributes to risky sexual behaviors among them as espoused by the AMT. It is recommended that fishers are targeted for alcohol use and risky sexual behavior programs and interventions since alcohol use is prevalent among them and most of them also engage in unprotected sexual intercourse after consuming alcohol.

**Supplementary Information:**

The online version contains supplementary material available at 10.1186/s12889-023-16239-w.

## Background

Alcohol consumption is part of human social behavior and constitutes a routine part of social life in many countries [1]. Current estimates indicate that globally, on average, 2.3 billion people consume 32.8 g of alcoholic beverages per day [2], and strong evidence also abounds that situation in the African region is even higher by about 20 percent, amounting to 40.0 g per day [2].

Although most of Ghana's population abstains from alcohol use [3], it is becoming a significant public health concern. For instance, the amount of alcohol consumed in Ghana has increased over time. According to the Ghana NCD Alliance [3], alcohol consumption per person in Ghana increased from 1.46 L per person in 1960 to 2.7 L per person in 2016. Also, between 2010 and 2019, the consumption of pure alcohol in Ghana increased from approximately 29 million liters to 40.2 million liters [4]. Excessive alcohol intake can lead to fatal traffic accidents, damage vital human organs such as the liver, and heart, and contributes to depression and violence [5, 6].

Alcohol consumption may be harmful but has some health benefits. According to the National Institutes of Health [7], moderate alcohol intake is seen as good for the heart and circulatory system, whilst heavy drinking, on the other hand, is seen as a major cause of preventable deaths in most countries.

Prior studies have found over-indulgence of fishers in alcohol in fishing communities [8–10]. The study area, Elmina, is also noted to be a fishing community where most fishers engage in excessive use of alcohol [11]. Varied reasons have been documented for the excessive use of alcohol amongst fishers in fishing communities. Studies by Anarfi [12] and Caldwell et al. [13] reported that fishers face several challenges, including housing challenges, financial problems, isolation and loneliness, and frustrations, which leads many of them to take to excessive drinking of alcohol at fishing communities points as a coping mechanism. Also, Tumwesigye et al. [9] explained that some fishers consume alcohol to fight off the stench and odor whilst fishing. Other studies have also attributed alcohol abuse among fishers as a coping mechanism against exposure to the risk of their work at sea [14, 15]. These explanations serve as evidence of alcohol abuse among fishers in fishing environments.

Earlier studies have identified alcohol consumption as one of the major drivers of sexual risk behaviors in fishing communities [9, 16, 17]. Tumwesigye et al. [9] highlighted that excessive alcohol use contributes to multiple sexual relationships, sex with non-regular partners, engagement in transactional sex, and inconsistent and incorrect use of condoms. Studies on alcohol use have found excessive use of alcohol among fishers in many fishing communities [10]. Studies by FAO [18] and Setiawan et al. [15] found frequent mobility and high levels of alcohol consumption among fishers before and during sexual encounters as a factor in their engagement in unsafe sexual practices. According to Tumwesigye et al. [9] high HIV risk exposure in fishing communities is frequently attributed to risk-taking behavior among fishers, including excessive alcohol use and risky sexual behavior. Tumwesigye et al. [9] and Gerbi [19] found that the likelihood of unprotected sex with multiple sexual partners increases significantly when people are influenced by excessive alcohol. In effect, alcohol consumption contributes to a higher probability of having unprotected sex and to a higher risk of HIV infection.

Few studies in sub-Saharan Africa, including Ghana, have examined alcohol consumption among fishers [9, 20, 21] and explored how alcohol consumption influences the risky sexual behavior of fishers [9, 22, 23]. Thus, there is a dearth of studies on alcohol consumption and risky sexual behavior among fishers. Examining how alcohol consumption influences the risky sexual behavior of fishers will help policymakers and researchers to design appropriate public policies and education initiatives to reduce risky sexual behavior among fishers. This study, therefore, investigated alcohol consumption, predictors of alcohol consumption, and the reasons for drinking alcohol among fishers in Elmina. It also examined fishers’ engagement in sex after alcohol consumption, the use of condoms with sexual partners after drinking alcohol, and predictors of the use of condoms with sexual partners after drinking alcohol.

### Theoretical framework

The theoretical framework that guided this study was the Alcohol Myopia Theory (AMT). According to AMT [24], consuming too much alcohol has both mental and physical effects which causes a person to act mentally short-sighted. Thus, the judgment of intoxicated people on situations may be compromised since they tend to make short-sighted decisions. In addition, the ability of intoxicated people to comprehend a wide range of information are limited [25], which further compromise their judgements on situations.

Excessive alcohol consumption can occasionally result in unexpected actions such as risky sexual behaviors. Intoxicated people lack the mental capacity to evaluate the benefits and risks of a particular course of action. Alcohol intoxication among fishers is common and it influence their risky sexual behaviors such as having multiple sexual partners and engaging in unprotected sex [9, 26]. In this study, we used the AMT to explain the phenomena of fishers engaging in sex after alcohol consumption, and condom use with sexual partner(s) after alcohol consumption.

## Methods

### Study design and sampling procedure

**Fig. 1:**
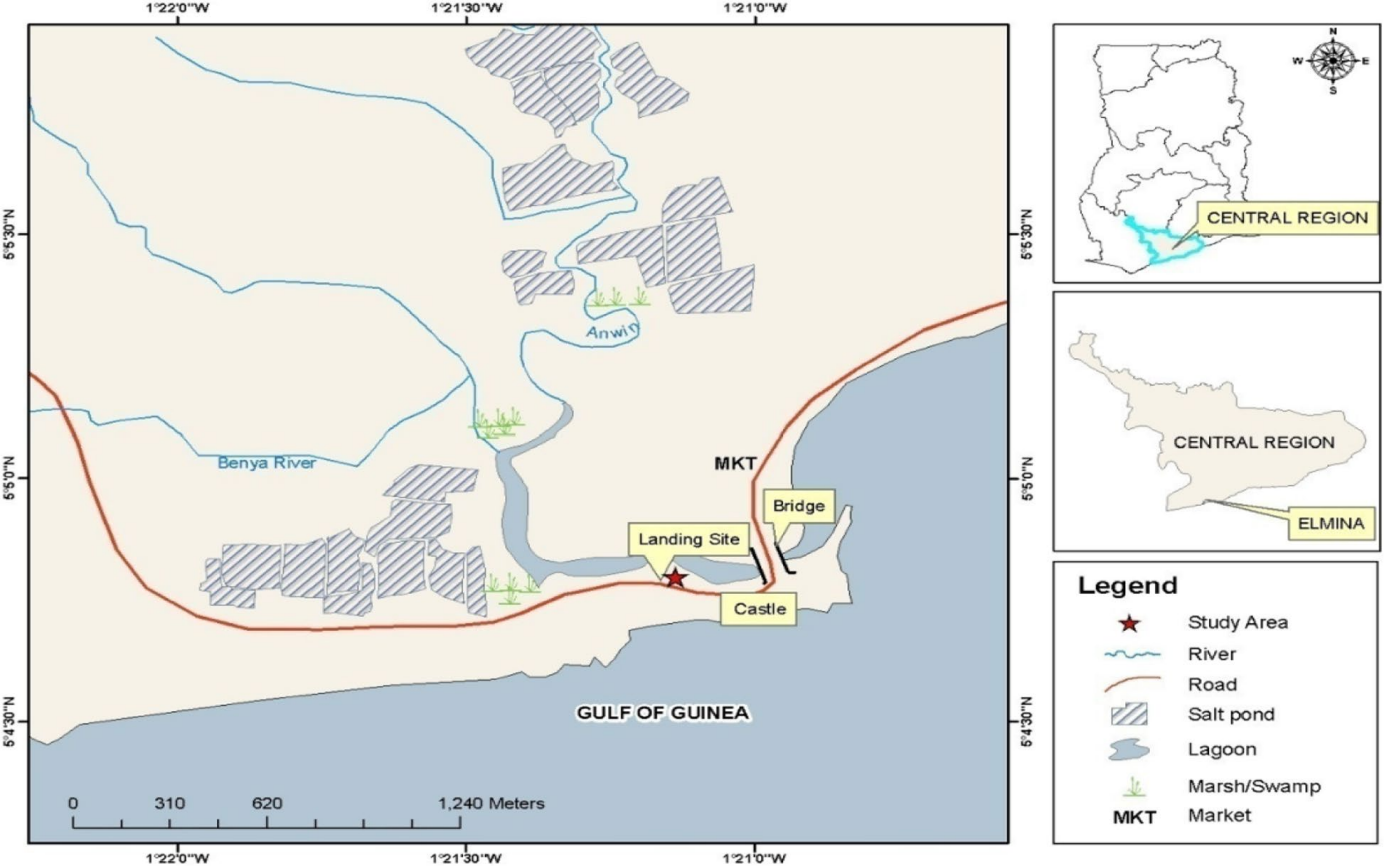
Map of Study Area in KEEA Municipal Assembly Source: Fieldwork, July–August 2017

This study used a cross-sectional convergent parallel mixed-method design to collect data from artisanal fishers in Elmina in the Komenda Edina Eguafo Abirem (KEEA) Municipal Assembly in the Central region of Ghana (see Fig. 1). Convergent parallel mixed-method design is a mixed-method design where the researcher simultaneously collects qualitative and quantitative data, analyze these data separately and use the findings of both data to explain a social phenomenon [27–29]. Convergent parallel mixed-method design helps researchers compare or relate quantitative and qualitative data findings to understand a social phenomenon.

The population size of fishers in the study area is unknown, and consequently, the sample size for the quantitative study was calculated using the formula:

$$n=\frac{{\left(Z-score\right)}^{2}*StdDev\ \left(1-StdDev\right)}{{\left(Margin of error\right)}^{2}}$$

Where *n* = sample size, Z-score = 1.96 for level of confidence of 95%, Standard deviation (StdDev) = 0.5, and margin of error = 0.05.

$$n=\frac{{\left(1.96\right)}^{2}*.5\left(1-0.5\right)}{{\left(0.05\right)}^{2}}$$

$$n=384.16$$

The sample size calculation gave 384.16 participants and the figure was rounded up to 385 participants. Therefore, 385 participants were interviewed. Following the compilation of the list of fishers for each of the ten associations, a population of 690 fishers were identified through the listing process. A simple random sample by proportional allocation was employed to ensure that fishers in each of the ten associations had an equal probability of being chosen for inclusion in the study. The formula below was used to calculate the appropriate sample for each of the ten associations:

$$\mathrm{Fisher Association }\left(\mathrm{y}\right) = \frac{n\left(sample size\right)}{N\left(total population\right)}\times \mathrm{N}1\left(members in each association\right)$$

The sample size for each of the ten associations is shown in Table 1. Names of the 690 fishers were written on pieces of papers, and placed in ten containers. The names of the fishermen were then drawn at random from the containers. Only the expected sample size was chosen for each container.

**Table 1:** Sample Size Calculation

Name of Association	Population	Sample Size Estimated
Girls Fish Porters Group	74	41
Ayisa Fishmongers Association	100	56
Mpobeng Fish Traders Association	40	24
Canoe Fishers Association	80	44
Novisi Fish Mongers Association	60	34
Efar Batafo Fishers Association	80	45
Ghana Co-operative Fisheries Association	56	30
Boys ‘Hustlers’ Group	90	50
Elmina Canoe Owners Association	60	33
Konkohin Fishers Association	50	28
**Total**	**690**	**385**

The study used key informant interviews (KII) to obtain information from a group of individuals, and the subjects for the KIIs were selected using the purposive sampling method. Purposive sampling was employed because it involves a process for selecting research subjects based on their applicability to the study's point of view, research questions, and justification [30, 31]. The KEEA office was contacted by the authors to obtain information on the organizations working in the research area to promote sexual and reproductive health education for fishers. This information was used to compile a list of officials to be contacted. A community contact also provided names of individuals who were thought to have important knowledge about the study subject and could participate as opinion leaders. The contact person aided in securing participation in the KII for the leaders of the fishing associations. The information from the KEEA office and that from the community contact person led to the compilation of 30 key informants who participated in the KIIs (see Table 2). The subjects were chosen in a way that was universal, representative, and reliable since they made up a group that had the greatest and most relevant information needed for the research. The rationale of the KIIs was to learn more about the participants' perspectives on fishing, issues surrounding fishers' sexual behavior and its consequences, challenges they faced, and lessons they could apply in the future. Participants in the groups included officers from the KEEA Assembly office, directors of key government departments and units, and two members from each of ten fisher associations.

**Table 2:** Participants of the key informant interviews

Subject	Frequency
KEEA Administration Office	4
Municipal Health Directorate	1
Ghana AIDS Commission	1
Non-governmental or Community-based Organisations officials	2
Department of Fisheries	2
Community members (fisher associations, and other opinion leaders of the fishing community	20
Total	30

To complement the other data, focused group discussions were also utilized. Two FGDs were held with male and female fishers, each group comprising 10 participants. To properly provide information that would reflect the diversity of experiences on the research questions and the research, a varied sample was selected. For selecting the participants for the groups, age, residency status, and the capacity to provide in-depth information necessary to supplement the study questions were considered.

### Study setting

The study was conducted in Elmina, the administrative capital of the KEEA Municipality and the first European settlement in West Africa. Elmina is located in a harbor with a southern position on Ghana's Atlantic Ocean coast, 12 km West of Cape Coast, the regional capital for the Central Region (see Fig. 1). It was carved off from the Cape Coast Metropolis in 1988 and elevated to a Municipality in 2008 by Legislative Instrument (LI) 1857. The Municipality is bordered on the south by the Atlantic Ocean (Gulf of Guinea), east by the Cape Coast Metropolis, on the north by the Twifo-Hemang-Lower Denkyira District, and the west by the Mpohor-Wassa East District. The municipality has a total area of 452 square kilometers and a population density of 319.8 persons per square kilometer. Elmina is Ghana's second-largest fish landing point and is largely used by inshore boats and canoes. Two landing quays and a sizable fish market are also present [32].

### Measures

#### Type of fishing occupation

The term "fishing occupation" refers to economic activities which involves the capturing, processing, preserving, storing, transporting, selling of fish; fishing and fish related errands; and the repair, mending and maintenance of fishing related equipment and tools, such as boats, outboard motors, and fishing nets. The study classified types of fishing occupation into four; the fish catch group were the fishers who traveled on sea to catch fish, the post-harvest group engaged in activities such as processing, marketing, storage, transportation etc. of the fish. The maintenance and repair Group were those involved in the repair, mending and maintenance of boats, outboard motors, and fishing nets, whiles those engaged in the porterage of fish and running of errands were categorised as porters and errand group.

#### Alcohol consumption and alcohol consumption levels

Indulgence in alcohol consumption refers to the consumption of any alcohol drink. In this study respondents were asked “whether they consume any alcoholic drink?”. There was no timeline/timeframe for respondents’ consumption of alcohol. Also, alcohol consumption levels were measured as binge, moderate or cannot determine. Binge consumption means consumption of 5 or more alcoholic drinks during an occasion for men or 4 or more alcoholic drinks during an occasion for women [33], while moderate consumption means consumption of 1 alcoholic drink or less in a day for women or 2 alcoholic drinks or less in a day for men [34]. Cannot determine alcohol level means participant cannot determine the quantity of alcoholic drinks s/he took.

In this study, excessive alcohol consumption include binge consumption and consumption of 8 or more alcoholic drinks per week for women or consumption of 15 or more alcoholic drinks per week for men [33]. Adopting these standardized tools to measure alcohol consumption is a foundation for evidence-based assessment [35] and helps detect the levels of alcohol use and misuse [36].

#### Migration patterns

Generally, migration patterns refer to changes in the residence of fishers over time and the reasons for change in residence. The description of the migration patterns of fishers was beyond the scope of this study.

#### Living conditions

Living conditions refers to the factors that affect the way individual live in their environment, which influence their health and general wellbeing. These factors include access to portable water and sanitation facilities, and shelter. The description of the living conditions of fishers was beyond the scope of this study.

### Dependent variables

The dependent variables for this study were indulgence in alcohol consumption and whether a condom was used the last time respondent had sex after drinking alcohol. The responses were Yes and No.

### Independent variables

The independent variables for this study were the socio-demographic characteristics of participants. This included sex (male and female), age (< 25, 25–34, 35–44, and 45 +), education (no education, middle/JHS education, secondary/vocational and higher), ethnicity (Akan and non-Akan), years engaged in fishing activity (1–10, 11–20, 21–30, 31 +), and mobility status (mobile fisher and non-mobile fisher).

Religion was recategorized as no religion, Christian and non-Christian (Islam and African Traditionalist). Also, marital status was recoded as never married, currently married (married and cohabiting), and formerly married (divorced, separated and divorced). Type of fishing occupation was also recoded as fish catch group and non-fish catch group (post-harvest group, maintenance and repair group, and porters and errand group).

### Data collection

The data collection for this study was carried out between July and August 2017. A survey was conducted among 385 fishers using a semi-structured questionnaire. The semi-structured questionnaire covered varied topics, including mobility patterns, living conditions, risky sexual behaviors, and strategies for carrying out sexual reproductive health education (See Additional file).

Two focus group discussions (FGD) were conducted: one with male fishers and the other with female fishers, each consisting of 10 participants. The inclusion criteria were all artisanal marine water fishers aged 18 and above engaged in any form of fishing activity, such as actual fishing, fish porters, boat (canoe) repairers, fishing gear sellers, and fish traders. Anybody under the age of 18 who was found fishing in any form was excluded.

### Data analysis

The Statistical Package for Social Sciences (SPSS) version 25 was used to analyze the quantitative data. Descriptive statistics, specifically frequencies and percentages, were used to analyze the quantitative data. Pearson’s chi-square test was also used to examine two associations. First, the association between indulgence in alcohol consumption and type of fishing occupation. Second, the association between whether a condom was used the last time respondent had sex after drinking alcohol and type of fishing occupation. In addition, binary logistic regression was used to examine the predictors of alcohol consumption and predictors of condom use during last sexual intercourse after drinking alcohol. Binary logistic regression was used because the dependent variables (indulgence in alcohol consumption and whether a condom was used the last time respondent had sex after drinking alcohol) had dichotomous values, Yes (1) and No (0). All variables were statistically significant at *p* < 0.05.

The qualitative data were analyzed thematically. All interviews were audio-recorded and transcribed verbatim. We read all transcripts to gain a general understanding of participants' narratives. Statements/sentences relevant to the aims of the study were assigned codes. Codes that narrate similar experiences were grouped into basic themes. In addition, similar basic themes were grouped into organizing themes.

Rigor of findings are essential in qualitative research [37]. We engaged in two activities to ensure rigor of our findings. First, the qualitative data analysis was done by the second author. However, the analysis process and the themes that emerged were discussed with the first author and discrepancies were resolved and recorded. Second, the themes that emerged from the study were shared with colleagues with expertise in conducting qualitative research and analyzing qualitative data.

## Results

### Socio-demographic characteristics of participants

Table 3 shows the socio-demographic characteristics of participants interviewed for the survey. More than half of the 385 participants (51.4%) interviewed for the study were female. About 30 percent (29.9%) of the participants were adults aged 35 to 44, while 22 percent (22.3%) were aged below 25. More than half of the participants were mobile fishers (54.3%) and had attained Middle/JHS education (53.8%). Eight out of 10 participants were Christians, while less than one-tenth of participants (7.5%) had no religious affiliation. More than half of the participants (55.6%) were currently married, while about 3 out of 10 were never married. Four-fifth of participants (80.0%) belonged to the Akan ethnic group. One-fourth of participants (25.5%) were engaged in fish catch activities, while a little over one-fifth of participants (21.6%) were engaged in porters and errand activities. Furthermore, two-fifth of participants (40.5%) had worked 1–10 years, while one-fifth (20.0%) had worked for 21–30 years.

**Table 3:** Socio-demographic characteristics of respondents

Sex	Frequency	Percent
Male	187	48.6
Female	198	51.4
**Age**		
< 25	86	22.3
25–34	93	24.2
35–44	115	29.9
45 +	91	23.6
**Mobility Status**		
Mobile fisher	209	54.3
Non-mobile fisher	176	45.7
**Education**		
No education	129	33.5
Middle/JHS	207	53.8
Secondary/vocational and higher	49	12.7
**Religion**		
No religion	29	7.5
Christian	316	82.1
Islam	21	5.5
African Traditionalist	19	4.9
**Marital Status**		
Never married	112	29.1
Currently married	214	55.6
Formerly married	59	15.3
**Ethnicity**		
Akan	308	80.0
Non-Akan	77	20.0
**Type of fishing occupation**		
Fish catch group	98	25.5
Post-harvest group	149	38.7
Maintenance and repair group	55	14.3
Porters and errand group	83	21.6
**Years engaged in fishing activity**		
1–10	156	40.5
11–20	88	22.9
21–30	77	20.0
31 +	64	16.6

### Alcohol consumption among fishers

Participants were asked about their indulgence in alcohol consumption and the reasons for engaging in it. From Table 4, most participants (59.2%) indicated they engaged in alcohol consumption. A higher proportion of male (70.6%) than female (48.5%) participants reported indulging in alcohol consumption.

**Table 4:** Indulgence in alcohol, and drinking habits

	**Male (*n* = 187)**		**Female (*n* = 198)**		**Total (*n* = 385)**	
	Frequency	Percent	Frequency	Percent	Frequency	Percent
**Do you indulge in drinking alcohol?**						
Yes	132	70.6	96	48.5	228	59.2
No	55	29.4	102	51.5	157	40.8
Alcohol consumption levels*						
Binge	96	53.6	77	43.3	173	48.5
Moderate	74	41.3	62	34.8	136	38.1
Cannot determine	9	5.0	39	21.9	48	13.4

^*^ Total of alcohol consumption levels = 355

The study inquired whether participants were engaged in binge or moderate drinking habits. About half (48.5%) of participants who drink alcohol admitted to indulging in binge alcohol consumption. According to the data in Table 2, more males (53.6%) than females (43.3%) reported binge drinking. A little over 38 percent (38.1%) also said they consume alcohol in moderation, while 13.4 percent claimed they cannot determine.

Table 5 shows the association between indulgence in alcohol consumption and type of fishing occupation. From Table 5, a higher proportion of participants in non-fish catch group (63.4%) indulged in alcohol consumption than those in the fish catch group (46.9%). The association between indulgence in alcohol consumption and type of fishing occupation was statistically significant (*p* < 0.05).

**Table 5:** Association between indulgence in alcohol consumption and type of fishing occupation

**Variable**	**Indulgence in alcohol consumption**		
	**Yes** **Frequency (%)**	**No** **Frequency (%)**	***p*-value**
**Type of fishing occupation**			8.212**
Fish catch group	46 (46.9)	52 (53.1)	
Non-fish catch group	182 (63.4)	105 (36.6)	

*p* < 1 ∗ , *p* < 0*.*05 ∗  ∗ , *p* < 0*.*001 ∗  ∗  ∗

During the male and female FGDs, participants explained that due to the dangerous nature of fishing, many fishers refrain from alcohol to stay safe at sea. However, fishers tend to engage in excessive alcohol consumption when they return from fishing expeditions and their off days. Some participants made the following statements:

*There is time for everything. Alcohol is strictly forbidden on many of our canoes. We believe that our work is dangerous, so we advise our members not to drink on duty. I only ‘dissolve’ [consume alcohol] when I return from my fishing expedition. When I return home after fishing, I ‘dissolve’ a lot of Akpeteshie, Egya Appiah, and Joy Daddy [brand names of local alcoholic beverages in Ghana] with my friends in the drinking spots in town. (Male, FGD)*

*We usually don’t go to sea on Tuesdays, so we consider it our off day. We usually start our alcohol consumption on Monday evenings. But on Tuesdays, we have the whole day to play card games, draught, smoke, and drink a lot of alcohol. On Wednesdays, when you see us, you will not think we are the same men you saw on Tuesday who were drunk. (Male, FGD)*

Similarly, during the female FGD, participants narrated that although both male and female fishers consume alcohol, male fishers consume more alcohol than female fishers. They mentioned that, unlike the male fishers who gather and consume alcohol, it is not common to find female fishers who gather and consume alcohol. They elaborated that most female fishers consume alcohol with their boyfriends and spouses. The statement below emerged from a female FGD:

*Alcoholism is very common in fishing communities, but it is more common among male fishers who usually go fishing for weeks. They consume a lot of alcohol when they return from the fishing expedition. None of my female friends will go to the beer bar [pub] and buy alcohol. We often accompany our boyfriends or male friends to the beer bar [pub]. We often indicate beer and Akpeteshie [brand name of local alcoholic beverage in Ghana] when they offer to buy us alcohol. (Female, FGD)*

### Predictors of alcohol consumption

Table 6 shows the factors that predict alcohol consumption among fishers. The findings revealed that male fishers (AOR = 2.542; 95% CI = 1.554–4.158; *p*-value = 0.000), were more likely to consume alcohol. However, non-Christian fishers (AOR = 0.177; 95% CI = 0.055–0.573; *p* < 0.05), and fishers who belonged to fish catch group (AOR = 0.505; 95% CI = 0.303–0.841; *p* < 0.05) were less likely to consume alcohol.

**Table 6:** Binary logistic regression of predictors of alcohol consumption

**Variables**	**Nagelkerke R**^**2**^** = 0.162**		
	**AOR**	**95% CI**	**p-value**
**Sex**			
Male	2.542	1.554–4.158	0.000***
Female (RC)			
**Age**			
< 25	0.810	0.301–2.179	0.677
25–34	0.626	0.260–1.511	0.298
35–44	0.656	0.316–1.361	0.257
45 + (RC)			
**Education**			
No Education (RC)			
Middle/JHS	0.830	0.506–1.359	0.458
Secondary/vocational and higher	0.829	0.377–1.824	0.641
**Religion**			
No religion (RC)			
Christian	0.509	0.188–1.382	0.185
Non-Christian	0.177	0.055–0.573	0.004**
**Marital status**			
Never married (RC)			
Currently married	1.191	0.673–2.107	0.548
Formerly married	0.712	0.318–1.593	0.408
**Ethnicity**			
Akan	1.212	0.676–2.171	0.519
Non-Akan (RC)			
**Type of fishing occupation**			
Fish Catch Group	0.505	0.303–0.841	0.009**
Non-Fish Catch Group (RC)			
**Years engaged in fishing activity**			
1–10	1.363	0.571–3.253	0.486
11–20	2.014	0.839–4.834	0.117
21–30	2.048	0.923–4.544	0.078
31 + (RC)			
**Mobility status**			
Mobile Fisher	1.322	0.837–2.088	0.231
Non-Mobile Fisher (RC)			

*AOR* Adjusted odds ratio, *RC* Reference category; *p* < 1 ∗ , *p* < 0.05 ∗  ∗ , *p* < 0.001 ∗  ∗  ∗

### Perceived reasons for drinking alcohol

Participants were asked why they indulged in the consumption of alcohol. According to Table 7, most participants (58.5%) used alcohol to cope with their stress and forget about their problems, whereas 34.5 percent drank for pleasure. The smallest percentage of participants (7.0%) claimed they drank to improve their sexual performance or raise their self-confidence.

**Table 7:** Indulgence in drinking alcohol and reasons for drinking alcohol

	**Male (*n* = 133)**		**Female (*n* = 96)**		**Total (*n* = 229)**	
	Frequency	Percent	Frequency	Percent	Frequency	Percent
**Reasons for drinking alcohol**						
To cope with stress and forget about problems	65	48.9	69	71.9	134	58.5
For pleasure	60	45.1	19	19.8	79	34.5
Increase confidence/boost sexual performance	8	6.0	8	8.3	16	7.0

Three themes emerged from the qualitative data on reasons for alcohol consumption among fishers, which are similar to the quantitative findings: (a) drinking to kill loneliness and boredom, (b) forgetting family and work-related issues, and (c) drinking for fun.

### Drinking to kill loneliness and boredom

Participants narrated that many fishers are migrants from other fishing communities who stay temporarily in Elmina to trade. Incidentally, they come to the community without their families and friends, so to kill their loneliness and boredom, alcohol serves as an outlet. The following views were expressed during the FGDs:

*I don’t live in Elmina. I am only here to carry fish from the canoes when the fishers return from an expedition. My wife and other family members are not present with me. So, whenever I get the chance, I enjoy a drink [consume alcohol] with my fishing mates to relieve boredom and loneliness. (Male, FGD)*

*I came from Axim in the Western Region and presently lodge with friends in a market stall in the evenings. So, until the owner has closed from work, I have no place to rest or any form of entertainment, such as watching television. Many migrants’ fishers have accommodation challenges and so converge at the drinking bar [pub] where we can get a television to watch and get people to keep us company. Sometimes, we can spread our mat at the corner of this bar [pub] and sleep. We come to the bar [pub] not only to drink alcohol. But also to pass the time when you have no comfortable place to sleep or rest. (Male, FGD)*

### Drinking to forget family and work-related issues

Participants explained fishing does not yield a lot of money these days, making many fishers face financial hardships. They further explained that due to these hardships, many fishers could not meet their financial obligations at work and home, creating family and work-related issues. Consequently, they resort to drinking alcohol to take away their worries. Thus, drinking alcohol became a channel for venting their frustrations. Recounting their experiences, the undermentioned statements were shared:

*My brother, these days, we cannot go far into the sea to catch fish the way we used to because we cannot afford to purchase enough pre-mix fuel to allow us to go at a rate that will guarantee a larger fish catch. We all have financial difficulties, so when we get together, we talk about them over drinks to relieve some of our tension. (Male, FGD)*

*At home, our wives demand more feeding money, the children clamor for their school needs, and we constantly have debtors breathing down our necks. My brother, you must find a way to survive when things get difficult. Most of us utilise group drinking of alcohol as a coping mechanism to escape from our families' various expectations and high debt burdens. (Male, FGD)*

### Drinking for fun

Participants explained that most fishers tend to have money to spend when they return from their fishing expedition. They earn income from canoe owners after the sale of their fishing products. Consequently, they use their income to purchase alcohol and drink for fun with their peers. Below are views of fishers to buttress the theme:

*There is no time to have fun on the sea when we go fishing. Therefore, most fishers find it great fun to drink alcohol when they are not on any fishing expedition. (Male, FGD)*

*There is no interesting activity for us to indulge in, especially on Tuesdays when we don’t go to sea. There is no better fun than sitting, chatting, and having a few alcoholic drinks with your friends. (Male, FGD)*

### Engagement in sex after drinking alcohol

Table 8 presents the respondents' responses to the issue of whether they had ever engaged in sexual activity after consuming alcohol in the last 12 months. Most participants (64.0%) indicated that they have ever had sexual intercourse after consuming alcohol in the past 12 months. About 8 out of 10 female participants (79.2%) reported they have ever engaged in sexual intercourse after consuming alcohol in the last 12 months than male participants (53.0%).

**Table 8:** Ever had sexual intercourse after drinking alcohol in the last 12 months

	**Male (*n* = 132)**		**Female (*n* = 96)**		**Total (*n* = 228)**	
	Frequency	Percent	Frequency	Percent	Frequency	Percent
**Have you ever had sexual intercourse after drinking alcohol in the last 12 months?**						
Yes	70	53.0	76	79.2	146	64.0
No	62	47.0	20	20.8	82	36.0
Who was your sexual partner the last time you drank alcohol before sex? *						
Regular partner	26	37.1	38	50.0	64	43.8
Spouse	30	42.9	26	34.2	56	38.3
Casual partner	10	14.3	12	15.8	22	15.1
Commercial sex worker	4	5.7	0	0.0	4	2.8

^*^ Total of who was your sexual partner the last time you drank alcohol before sex = 146

Regarding whom participants had sex with the last time they consumed alcohol, most participants (43.8%) generally had sex with their regular partners (Table 8). By sex differentials, a higher proportion of female participants (50.0%) had sex with their regular partners the last time they drank, while most male participants (42.9%) had sex with their spouses the last time they drank. In addition, approximately 6% of male participants had sex with commercial sex workers the last time they drank.

The FGDs revealed that having sexual intercourse after drinking alcohol was common among fishers in the study area. Both male and female participants explained that they enjoy their sexual intercourse when they consume alcohol since it increases their libido.

*As for me, when I get ‘boozed’ [intoxicated], my sexual libido rises, and I must have a woman to satisfy my sexual need. Sex is more enjoyable when you, your partner, or both are drunk. (Male, FGD)*

*Anytime I drink alcohol with my partner, I have sex with him. I enjoy sex better when I am under the influence of alcohol. I get aroused when I consume alcohol. My partner knows this, and so he often offers me alcohol as it provides the opportunity for us to have sex. (Female, FGD)*

### Use of condoms with sexual partners after drinking alcohol

Table 9 shows that 7 out of 10 participants did not use a condom the last time they had sex after drinking alcohol. A higher proportion of female participants (76.3%) did not use a condom the last time they had sex after drinking alcohol than male participants (63.3%). The three main reasons why participants did not use a condom the last time they had sex after drinking alcohol were do not like using condoms (37.9%), forgetting to use a condom (33.0%), and having sex with a trusted regular partner (15.5%). The sexual act that happened too fast (1.9%) was the least reason participants did not use a condom the last time they had sex after drinking alcohol.

**Table 9:** Condom was used the last time respondent had sex after drinking alcohol

	**Male (*n* = 71)**		**Female (*n* = 76)**		**Total (*n* = 147)**	
	Frequency	Percent	Frequency	Percent	Frequency	Percent
**Whether a condom was used the last time respondent had sex after drinking alcohol**						
Yes	26	36.7	18	23.7	44	29.9
No	45	63.3	58	76.3	103	70.1
Reason for not using condoms last time of having sex after drinking alcohol*						
Forgot to use a condom	14	31.1	20	34.5	34	33.0
Do not like using condoms	19	42.2	20	34.5	39	37.9
My partner does not like to use condoms	3	6.7	2	3.4	5	4.9
Had sex with a trusted regular partner	8	17.8	8	13.8	16	15.5
Condom was not available	0	0.0	7	12.1	7	6.8
Sexual act happened too fast	1	2.2	1	1.7	2	1.9

^*^ Total of reasons for not using condoms last time having sex after drinking alcohol = 103

Table 10 shows the association between whether a condom was used the last time respondent had sex after drinking alcohol and type of fishing occupation. From Table 9, a higher proportion of participants in the non-fish catch group (34.2%) did not use a condom the last time they had sex after drinking alcohol than those from the fish catch group (16.7%). The association between whether a condom was used the last time respondent had sex after drinking alcohol and type of fishing occupation was statistically significant (*p* < 0.05).

**Table 10:** Association between whether a condom was used the last time respondent had sex after drinking alcohol and type of fishing occupation

**Variable**	**Whether a condom was used the last time respondent had sex after drinking alcohol**		
	**Yes** **Frequency (%)**	**No** **Frequency (%)**	***p*-value**
**Type of fishing occupation**			4.000**
Fish catch group	6 (16.7)	30 (83.3)	
Non-fish catch group	38 (34.2)	73 (65.8)	

*p* < 1 ∗ , *p* < 0*.*05 ∗  ∗ , *p* < 0*.*001 ∗  ∗  ∗

During the FGDs, participants were asked about their willingness and capacity to use condoms with sexual partners after consuming alcohol. Most male participants explained that their desire to use condoms worsens after drinking alcohol. Alcohol makes most of them more libido-driven, to the point that they either forget to wear condoms or refuse to do so even when they have some available. Participants reported the following:

*I have used condoms a couple of times, and in all those instances, I have not taken any alcohol. I never use condoms when I drink alcohol and I get drunk. My libido increases, and all I want is sex. The issue of sexual protection does not even come to mind. (Male, FGD)*

*I have had instances where I had sex without using condoms after drinking alcohol. Honestly, when I am alcohol-induced to have sex, I only realize the need for safe sex after the encounter. (Male, FGD)*

*Men don’t like using condoms, so if you have sex with a fisher after taking alcohol, there is no way he will agree to use it. Even when they are sober, our men do not like using condoms, so you can imagine when they are drunk. (Male, FGD)*

### Predictors of condom use during last sexual intercourse after drinking alcohol

Table 11 shows the factors that predict condom use during last sexual intercourse after drinking alcohol among fishers. The findings revealed that only ethnicity was a significant predictor of condom use during last sexual intercourse after drinking alcohol. Participants who belonged to the Akan ethnic group (AOR = 0.167; 95% CI = 0.053–0.527; *p* < 0.05) were less likely to use a condom during last sexual intercourse after drinking alcohol.

**Table 11:** Binary logistic regression of predictors of condom use during last sexual intercourse after drinking alcohol

	**Nagelkerke R**^**2**^** = 0.288**		
**Variables**	**AOR**	**95% CI**	***p*-value**
**Sex**			
Male	1.753	0.689–4.460	0.239
Female (RC)			
**Age**			
< 25	0.970	0.145–6.475	0.975
25–34	0.510	0.098–2.652	0.423
35–44	1.651	0.403–6.769	0.486
45 + (RC)			
**Education**			
No Education (RC)			
Middle/JHS	1.062	0.372–3.032	0.910
Secondary/vocational and higher	1.269	0.334–4.821	0.726
**Religion**			
No religion (RC)			
Christian	0.211	0.040–1.109	0.066
Non-Christian	0.082	0.004–1.553	0.096
**Marital status**			
Never married (RC)			
Currently married	1.072	0.334–3.445	0.907
Formerly married	0.600	0.104–3.458	0.567
**Ethnicity**			
Akan	0.167	0.053–0.527	0.002**
Non-Akan (RC)			
**Type of fishing occupation**			
Fish Catch Group	0.465	0.156–1.388	0.170
Non-Fish Catch Group (RC)			
**Years engaged in fishing activity**			
1–10	1.218	0.240–6.189	0.812
11–20	2.733	0.566–13.191	0.211
21–30	0.291	0.049–1.724	0.174
31 + (RC)			
**Mobility status**			
Mobile Fisher	0.680	0.275–1.682	0.404
Non-Mobile Fisher (RC)			

*AOR* Adjusted odds ratio, *RC* Reference category; *p* < 1 ∗ , *p* < 0.05 ∗  ∗ , *p* < 0.001 ∗  ∗  ∗

## Discussion

This study investigated alcohol consumption among fishers in Elmina, predictors of alcohol consumption, and why they consume alcohol. In addition, we explored fishers' engagement in sex after alcohol consumption, their use of condoms with sexual partners after consuming alcohol, and predictors of the use of condoms with sexual partners after drinking alcohol.

The study found that 59.2% of fishers indulge in alcohol consumption. The prevalence of alcohol consumption among fishers (59.2%) in this study is higher than the prevalence of alcohol consumption reported among the general population in Ghana. For instance, Ghana NCD Alliance [3] found that the prevalence of alcohol consumption among the general population was 47.4% as of 2022. This finding is not surprising since studies have documented over-indulgence of fishers in alcohol [8–10].

The findings also showed that most fishers in Elmina indulged in binge consumption of alcohol, especially when they return from their fishing expeditions and on their off-days. The prevalence of binge consumption of alcohol among fishers (48.5%) in this study is higher than the prevalence of binge alcohol consumption reported among the general population (9.4%) in Ghana as of 2022 [3]. This finding supports earlier studies which reported higher alcohol consumption as a common habit amongst fishers [9, 16, 17]. For instance, Wickremasinghe et al. [20] study in three fishing communities in Sri Lanka found that alcohol consumption ranged between 65 and 88% among fishers. A study by Kher [8] found that when fishers are paid on their return from the fishing expedition, they tend to spend most of their money on alcohol and women. According to the AMT, drinking alcohol can make people more likely to feel happy after becoming intoxicated. It is not unexpected to learn that fishers enjoy spending excessive amounts of money on women and sex when they have extra money to spare [38]. This study recommends that policymakers, organizations, and health practitioners involved in alcohol use education and advocacy, should target fishers for alcohol use programs and interventions since binge alcohol consumption was prevalent among them.

Also, the study found that male fishers tend to consume more alcohol compared to female fishers. This finding resonates with earlier studies that have reported higher alcohol consumption among males than females [9, 39, 40]. For instance, Tumwesigye et al. [9] study in two fishing communities in Uganda found that a higher proportion of males (62.4%) got drunk in the past 30 days preceding the survey than females (52.0%). Since excessive alcohol consumption is associated with adverse health outcomes, such as depression and damaging vital human organs [5, 6], males are more likely to bear a disproportionate burden of these adverse health outcomes.

Regarding predictors of alcohol consumption among fishers, the study found that sex, religion and type of fishing occupation as significant predictors. Male fishers were more likely to consume alcohol. This finding collaborates with previous studies that found males are more likely to drink alcohol than females [41–43]. For instance, a survey by Ghana NCD Alliance [3] found that alcohol consumption was more prevalent among males than females. Sociocultural norms promote alcohol consumption among males [44, 45], while it frowns on alcohol consumption among females, and they are sanctioned when they indulge in excessive alcohol consumption [45].

Non-Christian fishers were less likely to consume alcohol than those without religious affiliations. A plausible explanation is that most religious groups frown on alcohol consumption among their members. For instance, in Islam, alcohol consumption is proscribed since it is perceived as Satanic, and therefore, it should be avoided by Muslims [46, 47].

With the type of fishing occupation, fishers who belonged to the fish catch group were less likely to consume alcohol than those who belonged to the non-fish catch group. A plausible explanation is that fishers who belong to the fish catch group are often on the sea plying their trade, and alcohol consumption may compromise their safety at sea and increase their risk of mortality. Therefore, they tend to consume no or less alcohol while fishing. Studies have linked alcohol consumption with work-related accidents and mortality among fishers [48, 49].

Both the quantitative and qualitative findings found that fishers consumed alcohol to kill their loneliness and boredom, to forget their family and work-related issues, and for pleasure. This finding support earlier studies' report of why fishers consume alcohol. For instance, studies have established that the desperation and frustration of being poor, and the inability to access financial capital for fishing expeditions, fosters reckless habits of excessive alcohol consumption among male fishers [12, 16]. Also, Caldwell et al. [13] explain that fishers face several challenges at their destination points, including housing challenges, isolation, and loneliness. Fishers, consequently, consume alcohol to escape from these challenges. According to the AMT, excessive alcohol use can occasionally lead to irrational feelings or acts that have detrimental consequences [24]. Some fishers drink a lot of alcohol because they believe that drinking and becoming intoxicated will make them less lonely, frustrated, and troubled.

In addition, the study found that most fishers have ever engaged in sexual intercourse after consuming alcohol. The prevalence of fishers who had ever engaged in sexual intercourse after consuming alcohol was higher than the prevalence reported in previous studies in Ghana. For instance, Abdul-Razak et al.’s [50] study among female adolescents in Sunyani Municipality in the Brong Ahafo Region of Ghana reported that a little over one-fifth (21.4%) of respondents engaged in sex after consuming alcohol and three out of five respondents who had sex after consuming alcohol used no condom during their sexual intercourse. Also, in a study by Aboagye et al.’s [51] among tertiary students in Hohoe Municipality in the Volta region, it was found that 35.1% of respondents identified unprotected sex as an effect of alcohol consumption.

Alcohol consumption tends to increase the libido of users [52], which could plausibly explain why fishers engage in sex after consuming alcohol. Although most participants had sex with their regular partners after consuming alcohol, a few had sex with commercial sex workers after consuming alcohol. This finding is similar to an earlier study by Sileo et al. [52], which found that drunk fishers patronize the services of commercial sex workers. The AMT construct, which contends that drunk persons lack the mental capacity to weigh the advantages and disadvantages of a given course of action, is supported by this research. Participants in a prior study in fishing communities, for example, claimed that they never consider using condoms even when having intercourse with irregular partners like commercial sex workers when heavily intoxicated after consuming a lot of alcohol [53]. This is because the immediate rewards of risky activity, including sexual fulfilment, are frequently the attention-grabbing indicators most inebriated people are inclined to focus on.

Furthermore, most participants did not use a condom the last time they had sex after consuming alcohol. The dominant reasons for the non-use of condoms the last time fishers had sex after consuming alcohol were, do not like using condoms, forgetting to use condoms, and had sex with a trusted regular partner. Previous studies have cited not liking the use of condoms [54–56] and trusting sex partners [57, 58] as reasons for non-condom use. In addition, the likelihood of engaging in unprotected sex increase significantly when people are under the influence of alcohol [9, 19, 22, 59, 60]. Fishers' assertion that they do not use condoms even when they are intoxicated is a risky sexual behavior that puts them at risk of HIV and other sexually transmitted infections (STIs), especially when some indicate they engage commercial sex workers. Studies have linked alcohol consumption with an increased risk of HIV infection [61, 62]. For instance, a study in fishing communities in Uganda found that alcohol consumption contributed to 64% of new HIV infections in those communities [62]. When someone's judgment is impaired from alcohol consumption, poor decisions are made, such as not using condoms or using them inappropriately (Steele and Josephs, 1990). The AMT contends that after consuming large amounts of alcohol, a drinker's mental and emotional field of vision shrinks, causing them to make judgments that impair their ability to see the big picture and cause them to make decisions that are short-sighted [24, 63]. The results of risky sexual behavior suggest that most fishers in the study area are at risk of exposure to STIs due to their poor decisions after being intoxicated.

The study also found that only ethnicity was the significant predictor of condom use during last sexual intercourse after drinking alcohol among fishers. Fishers who belonged to the Akan ethnic group were less likely to use a condom during their last sexual intercourse after drinking alcohol than those who were non-Akan. We suggest that further qualitative studies be undertaken to explore the influence of ethnicity on the use of condoms during sexual intercourse after drinking alcohol. This knowledge will help researchers and policymakers create the best programs to educate and advise vulnerable ethnic groups on how to act after excessive alcohol drinking.

### Limitations of the study

This study has some limitations. First, the study was cross-sectional. Although cross-sectional studies help to understand alcohol consumption and risky sexual behaviors among fishers, a longitudinal study would have better enhanced our understanding of the nuances of alcohol consumption and risky sexual behaviors among fishers over time. Second, the study's findings cannot be generalized to all fishing communities in Ghana since they may have different contexts. Third, there was no timeframe/timeline for the consumption of alcohol by respondents, which is not robust. Despite these limitations, this study will help policymakers and researchers to design context-specific interventions to address alcohol consumption and risky sexual behaviors among fishers in Elmina.

## Conclusion

The AMT was used to analyze and explain the phenomena of fishers engaging in sex after alcohol consumption, and condom use with sexual partner(s) after alcohol consumption. The AMT provided insights into risky sexual behaviors among fishers who have consumed alcohol. According to this study, most fishers experienced a reduction in their mental and emotional field of vision after consuming high amounts of alcohol, which led them to make impulsive judgments, act against their better judgment, and take risks. This study revealed that alcohol consumption was prevalent among fishers, especially among male fishers. Although most fishers engaged in sexual intercourse after consuming alcohol, they did not use condoms. Fishers' non-use of condoms during sexual intercourse could increase their exposure to STIs, including HIV. The study also found that sex, religion and type of fishing occupation were significant predictors of alcohol consumption among fishers, while ethnicity was a significant predictor of condom use during last sexual intercourse after drinking alcohol.

Thus, program managers can use knowledge of AMT to predict risky behaviors among fishers and develop measures that will be effective in reducing some of the risky sexual behaviors frequently associated to alcohol consumption in fishing communities. Since excessive alcohol use and unprotected sexual activity after drinking alcohol are common among fishers, it is recommended that they should be given priority in alcohol-use and risky sexual behavior programs and interventions in the study area.

## Supplementary Information

**Additional file 1. **Questionnaire for fishers.

## Data Availability

The datasets generated and/or analyzed during the current study are available from the corresponding at reasonable request.

## Abbreviations

AIDS: Acquired Immunodeficiency Syndrome
ECH: Ethics Committee for the Humanities
FAO: Food and Agriculture Organization
FGD: Focus Group Discussion
HIV: Human Immunodeficiency Virus
JHS: Junior High School
KEEA: Komenda Edina Eguafo Abirem
LI: Legislative Instrument
SPSS: Statistical Package for Social Sciences
WHO: World Health Organization
