# Supplementary material for: Alcohol consumption and risky sexual behaviors among fishers in Elmina in Ghana
Source: BMC Public Health. 2023 Jul 11;23:1328. doi: 10.1186/s12889-023-16239-w (PMC10337065; doi:10.1186/s12889-023-16239-w)
Supplement: Supplementary file 1 — Additional file 1. Questionnaire for fishers. [file 12889_2023_16239_MOESM1_ESM.docx]

**QUESTIONNAIRE FOR FISHERS**

**SECTION A: SOCIO-DEMOGRAPHIC BACKGROUND**

To start I would like to ask some questions about yourself:

| DB1 | How old are you today? | 1. <25 [ ] 2. 25-34 [ ] 3. 35-44 [ ] 4. 45- 54 [ ]  5. 55-64 [ ] 6. 65-74 [ ] 7. 75+ 99. Don’t know |  |
| --- | --- | --- | --- |
| DB2 | What is your sex? | 1. Male [ ] 2. Female [ ] |  |
| DB3 | What is your highest educational level achieved? | 1. Never been to school [ ] 2. Primary [ ] 3. JSS/JHS [ ] 4. Middle School [ ] 5. SSS/SHS [ ]  6. Vocational/Technical/Commercial [ ]  7. Post-Middle/Post-Secondary Cer [ ]  8. Post-Secondary Diploma [ ] 9. Post-Graduate 9. Other(specify) |  |
| DB4 | What is your religion? | 1. Islam [ ] 2. African Traditionalist [ ] 3. No Religion [ ] 4. Christianity [ ] 5. Other (specify) |  |
| DB5 | Marital status | 1.Single [ ] 2. Cohabiting/informal/Consensual [ ]  3. Married [ ] 4. Divorced [ ] 5. Separated [ ]  6. Widowed [ ] |  |
| DB6 | Which type of fishing activity do you  engagein? | 1. Fish catching [ ] 2. Post-harvesting [ ] 3. Maintenance and Repair [ ] 4. Porters and errands [ ] 5. Other (specify) |  |
| DB7 | How long have you being engaged in this  activity in this community? | ………………………………………………. |  |
| DB8 | Nationality | 1. Ghanaian [ ] 2. Nigerian [ ] 3. Other ECOWAS states (specify) …………… 4. Africa, other than ECOWAS States (specify) 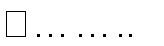 5. European (specify) ……… 6. Asian [ ] 7. Americas (North, South Caribbean) (specify)   ………..   1. Oceania (specify) …………………………… | **»**→ DB9  **»**→ MS1  **»**→ MS1  **»**→ MS1  **»**→ MS1  **»**→ MS1  **»**→ MS1  **»**→ MS1 |
| DB9 | Which ethnic group do you belong to? | 1. Fante [ ] 2. Other Akan [ ] 3. Ga Adangbe [ ]  4. Mole Dagbani [ ] 5. Ewe [ ] 6. Guan [ ]  7. Other (specify) ……  99. Don’t know [ ] |  |

###
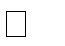
SECTION 5: RISKY SEXUAL BEHAVIOURS THAT EXPOSE FISHERS TO HIV

Now I want us to talk about your sexual activity in the last 12 months; whether you have had sex, whether you have had any sexual encounter (s) with any non-regular partner and the number of such partners. I would also ask you questions about condoms use in your sexual encounters within the period.

| RF1 | Have you had sex in the last 12 months? | 1.Yes | No |  |
| --- | --- | --- | --- | --- |
| RF2 | Have you had sexual intercourse with any non- regular partner in the last 12 months? | 1.Yes | No | IF ‘NO’  **»**→ RF4 |
| RF3 | If yes, about how many non-regular partners have you had intercourse with in the last 12  months | 1. One partner 2. Two partners  3. Three partners or more | |  |
| RF4 | Have you been using condoms in the last 12  months during sexual intercourse? | 1.Yes | 2. No | IF ‘NO’  **»**→ RF7 |
| RF5 | Was condom used the last time you had sex  with a non-regular partner? | 1.Yes | 2. No |  |
| RF6 | If yes, how often? | 1. Always 2. Not always  3.Occasionally 4. Never | |  |
| RF7 | IF no what was the main reason for not using a condom the last time you had sex with a non- regular partner? | 1. Partner objected to the use of condoms  2. Condom was not available  3. Trust partner/Didn’t think it was necessary  4. Attractiveness of partner | |  |
| RF8 | Have you heard of Fish-for-sex (FFS)  transactional relationship? | 1.Yes | 2. No | IF ‘NO’  **»**→ IP1 |
| RF9 | Have you ever engaged in Fish for sex? | 1.Yes | 2. No |  |
| RF10 | In the last 12 months have you engaged in FFS? | 1.Yes | 2. No |  |
| RF11 | Do you use a condom when you have FFS sex? | 1.Yes | 2. No |  |
| RF12 | If yes, what is the main reason for using a condom in FFS sex? | 1. Condom reduces pleasure of sex 2. My partner does not like to use condoms 3. Trust for partner/Didn’t think it was necessary   4. Condom not available | |  |
| RF13 | Did you use a condom the last time you had a  FFS sex? | 1.Yes | 2. No |  |
| RF14 | If yes what was the main reason for not using a  condom the last time you had FFS sex? | ……………………………………  …………………………. | |  |
| RF15 | Why do you engage in FFS transactional  relationship? | ……………………………………  …………………………. | |  |

###
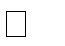
SECTION 5: ALCOHOL RELATED QUESTIONS

| AR1 | Do fishers in the community indulge in drinking alcohol? | 1.Yes | 2. No |  |
| --- | --- | --- | --- | --- |
| AR 2 | If yes, how would you describe the drinking habits? | 1. Excessive 2. Moderate  3. Occasional | |  |
| AR 3 | What are the reasons for fishers to engage in excessive drinking? | 1. To release stress and forget about problems 2. To have fun 3. To increase confidence to pursue sexual opportunities 4. It’s a work culture 5. To improve sexual performance | |  |
| AR 4 | Do you take any alcoholic drink? | 1. Yes 2. No | |  |
| AR 5 | If yes, how would you describe the drinking habits? | 1. Weekly  2. Daily  3. Monthly | |  |
| AR 6 | If yes, how many do you drink? |  | |  |
| AR 7 | Which type of alcohol have you mostly drunk during the last 4 weeks? | 1. Beer  2. Spirits (whisky, brandy, etc)  3. Palm wine  4. Traditional brew | |  |
| AR 8 | Which type of alcoholic drink did you have the last time you took an alcoholic drink? | 1. Beer  2. Spirits (whisky, brandy, etc)  3. Palm wine  4. Traditional brew | |  |
| AR 9 | When do you usually drink alcohol? | 1. Daytime after work  2. Evenings after work  3. On Tuesdays when we don’t go for fish   1. During fish lean seasons 2. At festive events, a party 3. After a fishing expedition | |  |
| AR 10 | Who do you usually drink alcohol with? | 1. Other persons I meet in the bar  2. Friends/workmates  3. People I meet at the bar  4. Girlfriends | |  |
| AR 11 | What is the main reason you drink alcohol | 1. To relax  2. To feel more open  3. To have fun  4. To increase my confidence to pursue sexual opportunities  5. The people I am with drink  6. I am stress about personal life  7. Because I am angry or irritable  8. Because UI am sad or lonely  9. I quarreled with my friends/relative/partner  10. Because I wasn’t to escape or forget problems  11. To face dangers at sea  12. To improve sexual performance | |  |
| AR 12 | Have you ever had sexual intercourse after drinking alcohol? | 1. Yes 2. No | |  |
| AR 13 | If yes, who was your sexual partner the last time you drank alcohol and before sex? | 1. Spouse 2. Regular girlfriend/boyfriend 3. Casual partner   5. Can’t ecall | |  |
| AR 14 | How frequently do you drink alcohol and then have sexual intercourse? | 1. Never 2. Sometimes 3. Most times 4. Always | |  |
| AR 15 | In the last 4 weeks how many of your sexual partners did you meet after drinking alcohol? | 1. None 2. 1 partner 3. 2 partners 4. 3 or more partners 5. Not sure | |  |
| AR 16 | Did you use a condom the last time you had sex after drinking alcohol? | 1. Yes 2. No | |  |
| AR 17 | What was the main reason for not using a condom the last time you had sex after drinking alcohol? | 1. Forgot to use condom 2. Don’t like using condoms 3. My partner does not like to use condoms 4. Trust partner 5. Condom was not available 6. Sexual act happened too fast | |  |
| AR 18 | In the last 4 weeks how often have you used a condom when having sex after drinking alcohol? | 1. Always 2. Not always 3. Occasionally/sometimes 4. Not often 5. I don’t remember 6. Never | |  |
| AR 19 | Do you keep condoms on you? | 1. Yes 2. No | |  |
| AR 20 | If yes, why? |  | |  |
| AR 21 | Are condoms readily available in the drinking places in the community? | 1. Yes 2. No | |  |
| AR 22 | Would you like to see condoms made available at alcohol consumption venues (bars)? | 1. Yes 2. No | |  |
| AR 23 | Would you use condoms made available at alcohol venues? | 1. Yes 2. No | |  |

**INTERVIEW GUIDE**

1. Describe the drinking habits of fishers in the Elmina fishing community?
2. How are the drinking sessions organised?
3. Where do people drink in the fishing community and why?
4. What reasons account for alcohol consumption among fishers in the fishing community?
